# Supplementary material for: Multiomics Reveals the Effect of Root Rot on Polygonati Rhizome and Identifies Pathogens and Biocontrol Strain
Source: Microbiol Spectr. 2022 Feb 28;10(2):e02385-21. doi: 10.1128/spectrum.02385-21 (PMC9045327; doi:10.1128/spectrum.02385-21)
Supplement: SUPPLEMENTAL FILE 9 — Supplemental material. Download SPECTRUM02385-21_Supp_1_seq10.pdf, PDF file, 0.9 MB [file spectrum02385-21_supp_1_seq10.pdf]

Additional files

Additional files 1 (Supplementary Figure 1-5)

Figure S1. Sampling diagram in this study. **Figure S2**. Transcriptome analysis of all healthy and diseased plant samples. **Figure S3**. Bacterial and fungal communities of all healthy and diseased plant samples. **Figure S4**. Bacterial and fungal communities and differential analysis of zone (ZS) and furrow soil (FS) in all plants. **Figure S5**. Bacterial and fungal communities and differential analysis of healthy and diseased rhizome samples. **Figure S6**. Function and phenotypic prediction of rhizosphere bacteria.

Additional files 2 (Table. S1-7)

Table S1. Polysaccharide and saponin content and fresh weight of healthy and diseased plants. **Table S2**. DEGs, GO and KEGG enrichment of pathways. **Table S3**. OUT and sequences numbers of 16S and ITS. **Table S4**. Alpha diversity index table. **Table S5**. Adonis and Anosim analysis of different groups. **Table S6**. Significance test between groups. **Table S7**. Predicted phenotypic differences between groups. **Table S8**. Isolated fungi and bacteria from diseased and healthy rhizomes, respectively.

Additional files 3 (Supplementary Method 1-3)

Method S1 and S2: *P. cyrtonema* rhizome polysaccharide and saponin metabolites profiling. **Method S3:** Bioinformatic and statistical analysis.

Additional files 1 (Supplementary Figure 1-6)

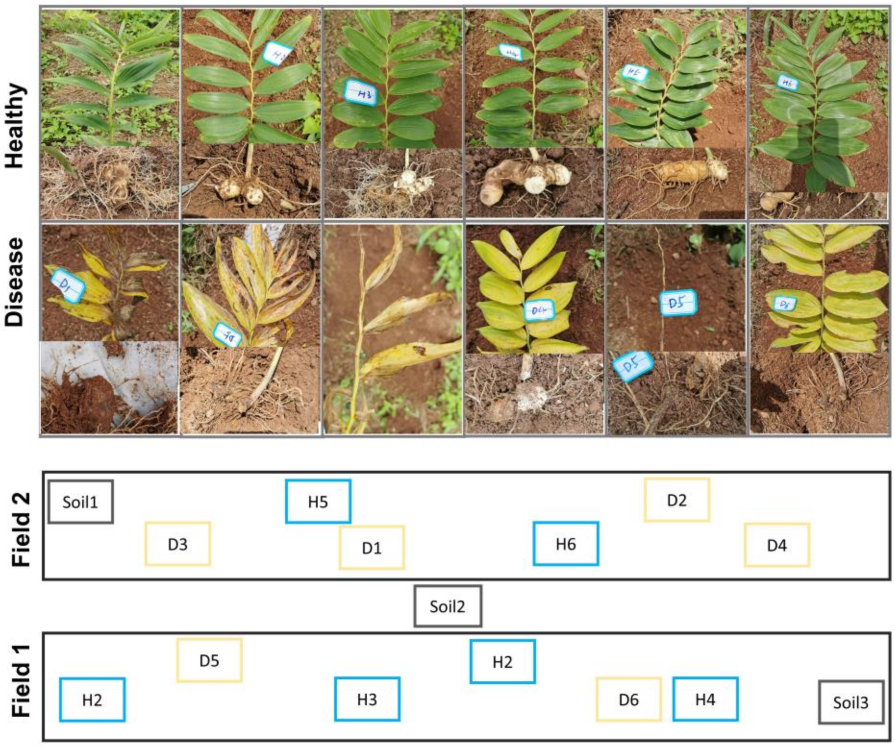

Fig. S1 Sampling diagram in this study. H1-5: healthy samples; D1-6: diseased samples; Soil1-3: no planted soil;

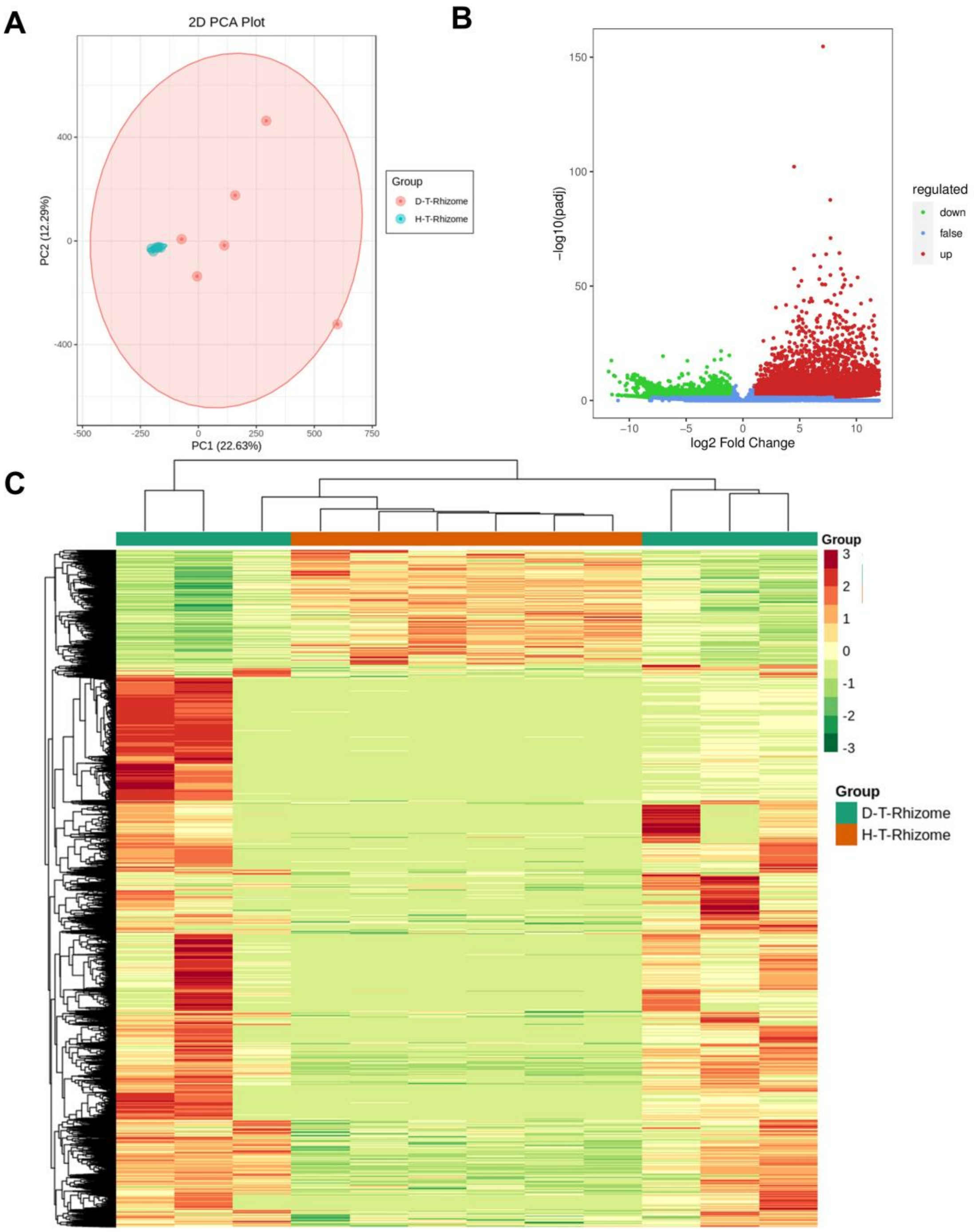

Fig. S2 Transcriptome analysis of all healthy and diseased plant samples. A PCA of rhizome transcriptome of the healthy and diseased rhizome; **B** Up and down gene in two samples; **C** Heat map clustering of DEGs in two samples.

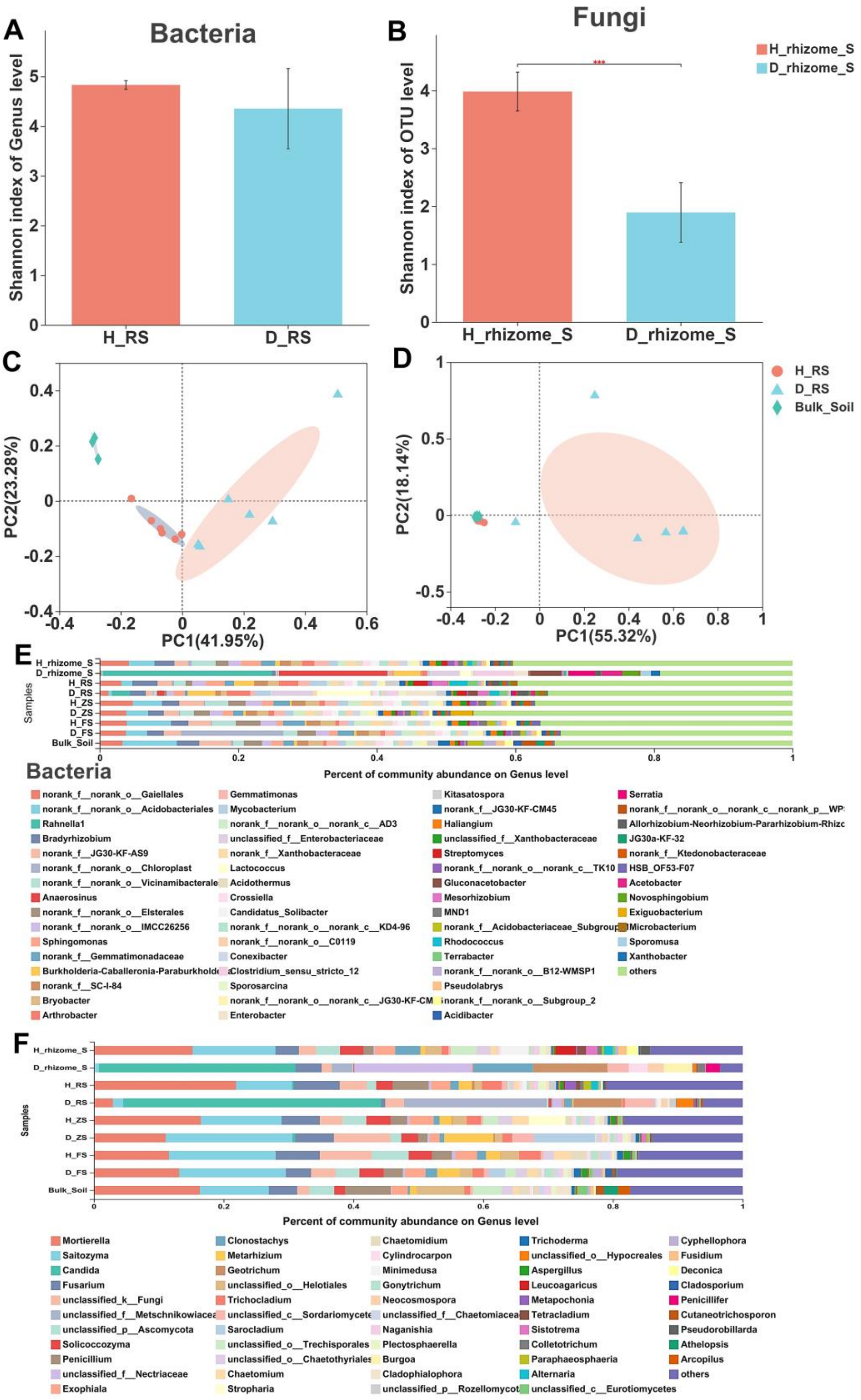

Fig. S3 Bacterial and fungal community of all healthy and diseased plant samples. A and B Alpha-Shannon diversity indices of bacterial and fungal communities of rhizosphere soil; **C and D** Principal component analysis (PCA) of bacterial and fungal communities beta diversity of rhizosphere soil; **E and F** Bacterial and fungal communities of all samples;

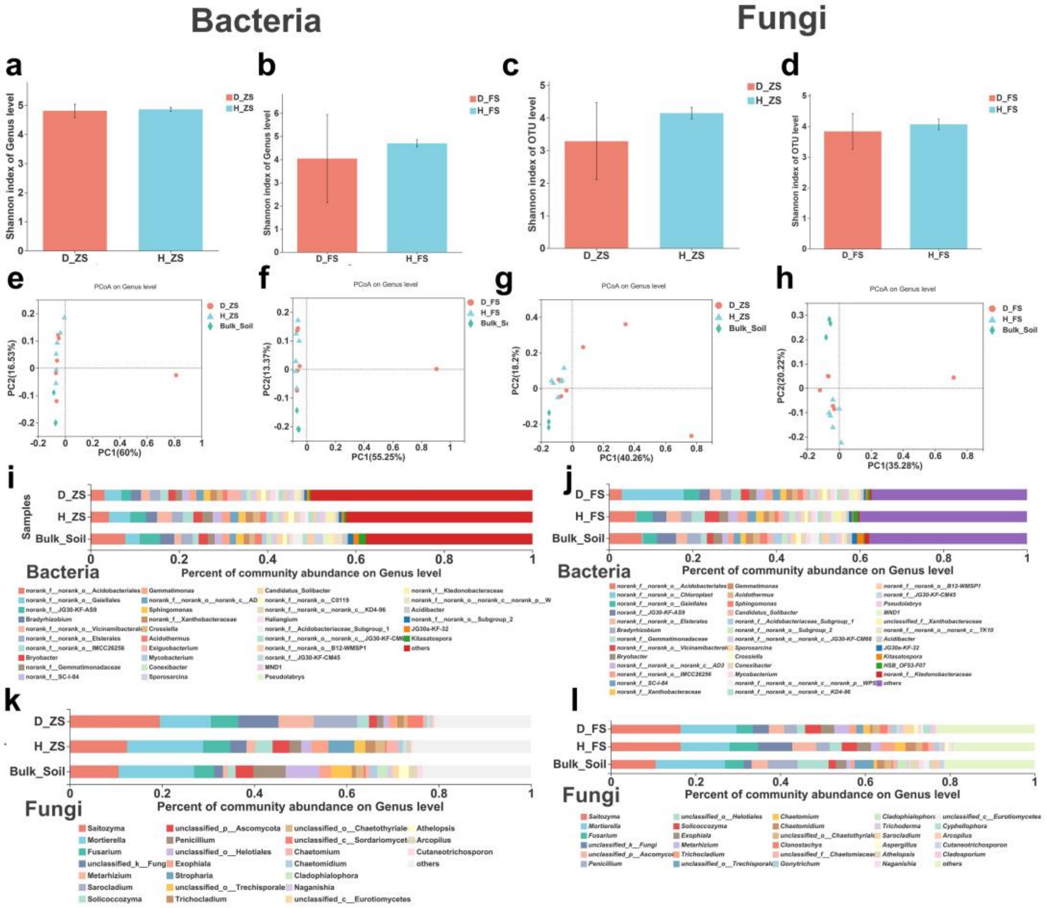

Fig. S4 Bacterial and fungal community and differentiate analysis of zone (D_ZS) and furrow soil (H_FS) in all plants. a-d Alpha-Shannon diversity indices of bacterial and fungal communities of all zone (ZS) and furrow (FS) samples; **e-h** Principal component analysis (PCA) of bacterial and fungal beta diversity; **i-l** Bacterial and fungal communities of zone and furrow soil.

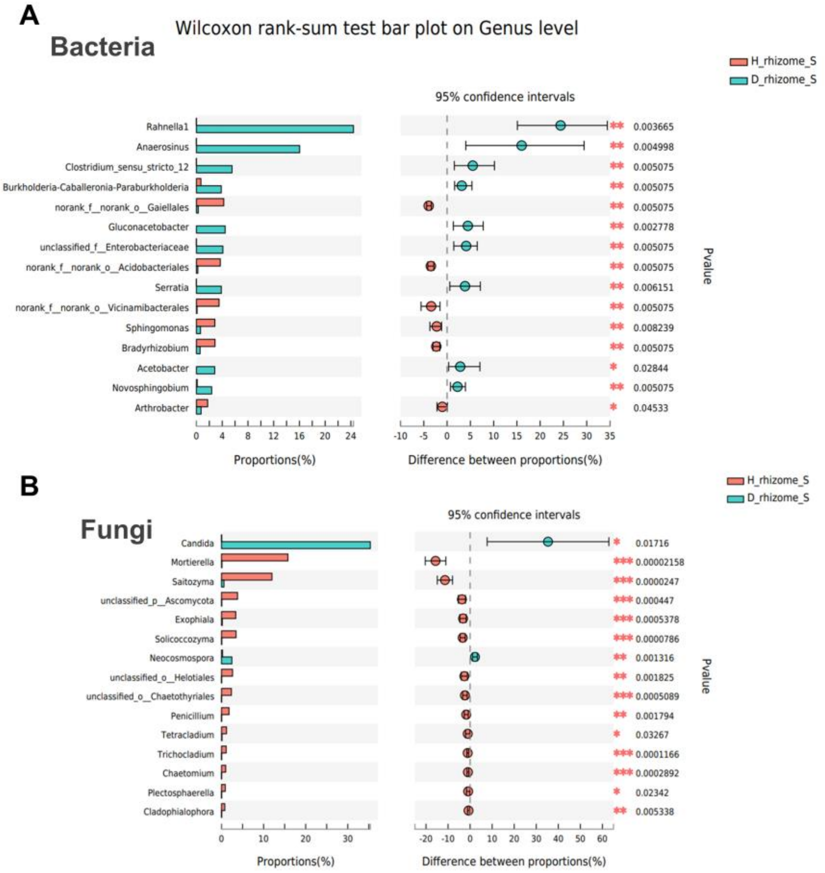

Fig. S5 Bacterial and fungal community and differentiate analysis of healthy (H_rhizome_S) and diseased rhizome (D_rhizome_S) samples.

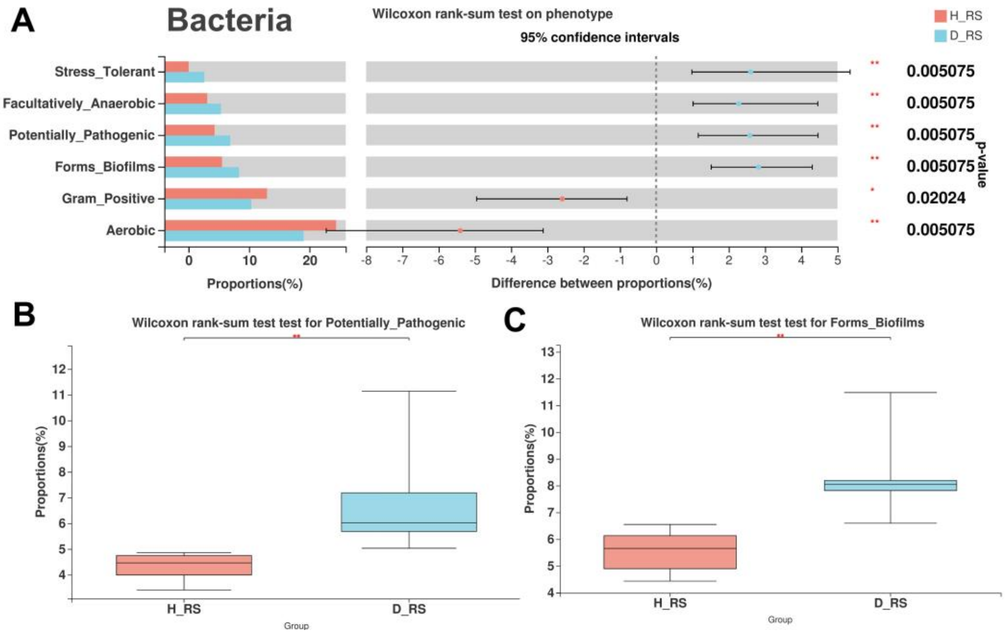

Fig. S6 Function and phenotypic prediction of rhizosphere bacteria of diseased (D_RS) and healthy (H_RS) samples.

Additional files 3 (Supplementary Method 1-3)

P. cyrtonema rhizome polysaccharide and saponin metabolites profiling

Supplementary Methods 1: saponin metabolites profiling

1.1 Sample preparation and extraction

Biological samples are freeze-dried by vacuum freeze-dryer (Scientz-100F). The freeze-dried sample was crushed using a mixer mill (MM 400, Retsch) with a zirconia bead for 1.5 min at 30 Hz. Dissolve 100 mg of lyophilized powder with 1.2 mL 70% methanol solution, vortex 30 seconds every 30 minutes for 6 times in total, place the sample in a refrigerator at 4°C overnight. Following centrifugation at 12000 rpm for 10 min, the extracts were filtrated (SCAA-104, 0.22 μm pore size; ANPEL,Shanghai, China, http://www.anpel.com.cn/) before UPLC-MS/MS analysis.

1.2 UPLC Conditions

The sample extracts were analyzed using an UPLC-ESI-MS/MS system (UPLC, SHIMADZU Nexera X2, https://www.shimadzu.com.cn/; MS, Applied Biosystems 4500 Q TRAP, https://www.thermofisher.cn/cn/zh/home/brands/applied-biosystems.html). The analytical conditions were as follows, UPLC: column, Agilent SB-C18 (1.8 µm, 2.1 mm * 100 mm); The mobile phase was consisted of solvent A, pure water with 0.1% formic acid, and solvent B, acetonitrile with 0.1% formic acid. Sample measurements were performed with a gradient program that employed the starting conditions of 95% A, 5% B. Within 9 min, a linear gradient to 5% A, 95% B was programmed, and a composition of 5% A, 95% B was kept for 1 min. Subsequently, a composition of 95% A, 5.0% B was adjusted within 1.1 min and kept for 2.9 min. The flow velocity was set as 0.35 mL per minute; The column oven was set to 40°C; The injection volume was 4 μL. The effluent was alternatively connected to an ESI-triple quadrupole-linear ion trap (QTRAP)-MS.

1.3 ESI-Q TRAP-MS/MS

LIT and triple quadrupole (QQQ) scans were acquired on a triple quadrupole-linear ion trap mass spectrometer (Q TRAP), AB4500 Q TRAP UPLC/MS/MS System, equipped with an ESI Turbo Ion-Spray interface, operating in positive and negative ion mode and controlled by Analyst 1.6.3 software (AB Sciex). The ESI source operation parameters were as follows: ion source, turbo spray; source temperature 550°C; ion spray voltage (IS) 5500 V (positive ion mode)/-4500 V

(negative ion mode); ion source gas I (GSI), gas II(GSII), curtain gas (CUR) were set at 50, 60, and 25.0 psi, respectively; the collision-activated dissociation(CAD) was high. Instrument tuning and mass calibration were performed with 10 and 100 μmol/L polypropylene glycol solutions in QQQ and LIT modes, respectively. QQQ scans were acquired as MRM experiments with collision gas (nitrogen) set to medium. DP and CE for individual MRM transitions was done with further DP and CE optimization. A specific set of MRM transitions were monitored for each period according to the metabolites eluted within this period.

Supplementary Methods 2: polysaccharide metabolites profiling

2.1 Chemicals and reagents

Methanol (MeOH) were purchased from Merck (Darmstadt, Germany). MilliQ water (Millipore, Bradford, USA) was used in all experiments. All of the standards were purchased from CNW (Shanghai Anpel), IsoReag (Shanghai) and TCI (Shanghai). The stock solutions of standards were prepared at the concentration of 2 mg/mL in MeOH. All stock solutions were stored at -20°C. The stock solutions were diluted with MeOH to working solutions before analysis.

2.2 Sample preparation and extraction

The freeze-dried materials were crushed using a mixer mill (MM 400, Retsch) with a zirconia bead for 1.5 min at 30 Hz. 20 mg of powder was diluted to a 500 μL with methanol: isopropanol: water (3:3:2 V/V/V), vortexed for 3 min and ultrasound for 30 min. The extract was centrifuged at 14,000 rpm under 4°C for 3 min. 50μL of the supernatant was mixed with 20 μL internal standard (ribitol, 100 μg/mL) and evaporated under nitrogen gas stream.The evaporated sample was transferred to the lyophilizer for freeze-drying. The residue was used for the further derivatization. The derivatization method was as follows: the sample was mixed with 100 μL solution of methoxyamine hydrochloride in pyridine (15 mg/mL). The mixture was incubated at 37°C for 2 h. Then 100 μL of BSTFA was added into the mixture and kept at 37°C for 30 min after vortex-mixing. The mixture was analyzed by GC-MS after diluting to an appropriate concentration.

2.3 GC-MS analysis

Agilent 7890B gas chromatograph coupled to a 7000D mass spectrometer with a DB-5MS column (30 m length × 0.25 mm i.d. × 0.25 μm film thickness, J&W Scientific, USA) was employed for GC-MS analysis of sugars. Helium was used as carrier gas, at a flow rate of 1 mL/min. Injections were made in the split mode with a split ratio 3:1 and the intection volume was 3 μL. The oven temperature was held at 170°C for 2min, and then raised to 240°C at 10°C/min , raised

to 280°C at 5°C/min , raised to 310°C at 25°C/minand held at the temperature for 4 min. All samples were analyzed in selective ion monitoring mode. The ion source and transfer line temperature were 230°C and 240°C, respectively.

Supplementary Methods 3: Bioinformatic and statistical analysis

3.1 PCA

Unsupervised PCA (principal component analysis) was performed by statistics function prcomp within R (www.r-project.org). The data was unit variance scaled before unsupervised PCA.

3.2 Hierarchical Cluster Analysis and Pearson Correlation Coefficients

The HCA (hierarchical cluster analysis) results of samples and metabolites were presented as heatmaps with dendrograms, while pearson correlation coefficients (PCC) between samples were caculated by the cor function in R and presented as only heatmaps. Both HCA and PCC were carried out by R package pheatmap. For HCA, normalized signal intensities of metabolites (unit variance scaling) are visualized as a color spectrum.

3.3 Differential metabolites selected

Significantly regulated metabolites between groups were determined by VIP ≥ 1 and absolute $\log_2$FC (fold change) ≥ 1. VIP values were extracted from OPLS-DA result, which also contain score plots and permutation plots, was generated using R package MetaboAnalystR. The data was log transform ($\log_2$) and mean centering before OPLS-DA. In order to avoid overfitting, a permutation test (200 permutations) was performed.

3.4 KEGG annotation and enrichment analysis

Identified metabolites were annotated using KEGG Compound database (http://www.kegg.jp/kegg/compound/), annotated metabolites were then mapped to KEGG Pathway database (http://www.kegg.jp/kegg/pathway.html). Pathways with significantly regulated metabolites mapped to were then fed into MSEA (metabolite sets enrichment analysis), their significance was determined by hypergeometric test's p-values.
